# A high-fat diet exacerbates arsenic toxicity in various organs. A systematic review of toxicity and mechanism

**DOI:** 10.1016/j.crtox.2025.100250

**Published:** 2025-07-16

**Authors:** Hoda Vahedi, Fateme Pourmotahari, Ali Akbar Oroojan, Amin Rasekhian, Soheila Alboghobeish

**Affiliations:** a Student Research Committee, Faculty of Medicine, , Tehran University of Medical Sciences, Tehran, Iran; b Department of Community Medicine, Faculty of Medicine, Dezful University of Medical Sciences, Dezful, Iran; c Department of Physiology, Faculty of Medicine, Dezful University of Medical Sciences, Dezful, Iran; d Student Research Committee, Faculty of Medicine, Dezful University of Medical Sciences, Dezful, Iran; e Department of Pharmacology, Faculty of Medicine, Dezful University of Medical Sciences, Dezful, Iran

**Keywords:** Arsenic toxicity, High-fat diet, Oxidative stress, Metabolic disorders, Organ damage, Systematic review, Environmental toxicology

## Abstract

**Background:**

Arsenic contamination in drinking water remains a primary global health concern. Recent evidence indicates that dietary habits, especially high-fat diets, may exacerbate arsenic-induced toxicity. This systematic review examines the impact of high-fat diets on arsenic toxicity across various organs and investigates the mechanisms underlying this interaction.

**Methods:**

A comprehensive literature search was conducted in PubMed, Scopus, Web of Science, and Embase databases. Studies involving animal models exposed to both arsenic and high-fat diets were included in this analysis. The methodological quality of the studies was assessed using the SYRCLE risk of bias tool.

**Results:**

A total of 20 studies met the inclusion criteria. High-fat diets were found to increase arsenic bioavailability and intensify oxidative stress in several organs, including the brain, heart, testes, and kidneys. Combined exposure to arsenic and high-fat diets resulted in complex metabolic disturbances, including impaired fatty acid metabolism in adipose tissue and a unique diabetogenic effect characterized by glucose intolerance without the typical features of type 2 diabetes. Interestingly, this combination also caused reduced weight gain despite increased insulin resistance and β-cell dysfunction. Modulation of hepatic autophagy pathways emerged as a possible mechanism linking arsenic toxicity to metabolic dysregulation.

**Conclusions:**

This review highlights that high-fat diets amplify arsenic toxicity through several mechanisms, including increased bioavailability, elevated oxidative damage, and altered metabolic signaling. The synergistic effects on glucose regulation and multi-organ toxicity underscore the importance of considering dietary habits in assessing arsenic-related health risks. Further research is warranted to clarify the molecular mechanisms involved and to design targeted strategies for mitigating arsenic toxicity, particularly in populations consuming high-fat diets.

## Highlights

• High-fat diets enhance arsenic toxicity in multiple organs.
• Co-exposure increases oxidative stress and metabolic disruption.
• Arsenic and high-fat diets impair glucose tolerance and β-cell function.
• Liver, brain, kidney, and reproductive systems are especially affected.
• Dietary factors should be considered in arsenic risk assessments.

## Graphical abstract

High-fat diets exacerbate arsenic-induced multi-organ toxicity through increased bioavailability, oxidative stress, and metabolic disruption.

**Figure:**
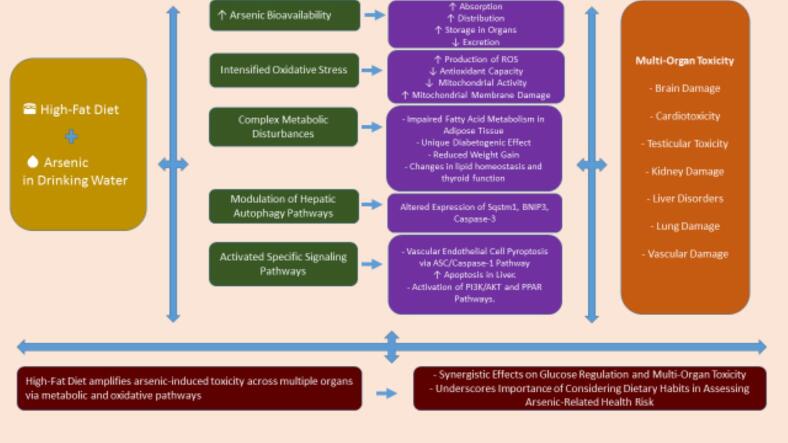

## Introduction

Arsenic (As) is a naturally occurring toxic metalloid found in various chemical forms, including inorganic arsenic (iAs), monomethyl arsenic (MMA), and dimethyl arsenic (DMA) (Chen and Costa, 2021). It is widely distributed in the Earth’s crust. It enters drinking water supplies through natural processes, such as leaching and erosion, as well as through anthropogenic activities, including mining and industrial discharge (Rathi and Kumar, 2021). High levels of arsenic in drinking water have been reported across numerous countries, including Iran, Bangladesh, China, India, Taiwan, Chile, Mexico, and the United States (Khosravi-Darani et al., 2022, Shokoohi et al., 2024, Murthy et al., 2024, Ganie et al., 2024). The World Health Organization (WHO) estimates that around 140 million people globally are exposed to arsenic-contaminated drinking water, which is considered the primary source of exposure for most populations (Dhane et al., 2024, Guo et al., 2024). Arsenic in water is primarily present as iAsIII and iAsV, which are more toxic due to their higher bioavailability compared to organic forms found in food (Frisbie and Mitchell, 2022, Shu et al., 2025).

Both acute and chronic exposure to arsenic pose serious health risks. Acute exposure may cause nausea, vomiting, abdominal pain, and neurological symptoms. In contrast, long-term exposure is linked to increased risks of skin, lung, bladder, and kidney cancers, as well as cardiovascular and reproductive disorders, including testicular atrophy and reduced spermatogenesis (Rajavardhan et al., 2021, Himeno and Hossain, 2021, Zhang et al., 2024, Adeogun et al., 2024).

Despite extensive research, the toxic effects of arsenic are still not fully understood, especially in the context of combined exposure with other environmental or lifestyle factors. While most studies focus on arsenic as a single toxicant, emerging evidence suggests that nutritional status and dietary patterns significantly modulate arsenic toxicity (Zeng et al., 2021, Sijko and Kozłowska, 2021).

The global rise in obesity, fueled by the widespread consumption of high-fat and processed foods, has created a new context in which arsenic exposure may be more harmful. Approximately 1.6 billion adults worldwide are classified as overweight or obese (Sørensen et al., 2022, Chen et al., 2024). Recent studies indicate that high-fat diets and obesity may increase arsenic bioaccumulation in various organs, leading to exacerbated toxic effects, including oxidative stress and metabolic disturbances (Calderón-DuPont et al., 2023, Bibha et al., 2024).

Currently, arsenic regulatory standards—such as the WHO and U.S. EPA limit of 10 µg/L—are primarily based on epidemiological data from healthy adult populations (Frisbie and Mitchell, 2022). These standards may fail to account for vulnerable subgroups, such as obese individuals with high-fat dietary intake.

This systematic review aims to evaluate the effects of high-fat diets on arsenic-induced toxicity in various organs and to explore the biological mechanisms underlying this interaction.

## Materials and methods

### Protocol

PRISMA guidelines directed our study selection, screening, and data extraction processes (Page et al., 2021).

### Eligibility criteria

We included in vivo animal studies in which subjects were exposed to both arsenic and a high-fat diet (HFD), with comparison groups exposed to either one or neither. Studies focusing solely on these factors or other dietary or pharmacological interventions for arsenic toxicity were excluded.

### Study design

Our restrictive criteria defined simultaneous and chronic arsenic exposure in drinking water alongside an HFD, specifically diets providing over 40 % of calories from fat.

### Searching methods and study selection

A systematic search was conducted in PubMed, Scopus, Web of Science, and Embase without time restrictions and limited to English-language articles. The final search was conducted on September 14, 2024. Search strategies were developed using MeSH, Emtree, and free-text terms related to arsenic, high-fat diets, and toxicity (Bramer et al., 2018). The full search strings for each database, including Boolean operators and filters, are provided in Appendix 1, as per the PRISMA guidelines. Additional articles were identified through manual reference checks (Chandler et al., 2019, Teixeira et al., 2024). After duplicate removal in EndNote, titles and abstracts were screened independently by two reviewers (SA and HV); discrepancies were resolved by a third reviewer (AAO). Study selection was based on the PICO framework (de Sousa et al., 2024).

### Data extraction

Data were extracted using a predefined form, which captured the first author's name, publication year, sample size, sampling type, study population, aims, experimental methodology (in vivo, ex vivo, or in vitro), key findings, language, gender, age, and study type.

### Quality assessment

Two authors independently assessed the risk of bias using the SYRCLE risk of bias tool (Hooijmans et al., 2014), adapted from Cochrane's tool for clinical studies to animal studies (Higgins et al., 2011). This tool comprises 10 questions covering selection, performance, detection, attrition, reporting, and other biases. Questions were answered “YES” (adequately addressed), “NO” (not addressed), or “Unclear” (insufficient information). Based on these answers, the risk of bias domains was classified as low, high, or unclear.

## Result

### Search results

**Fig. 1:**
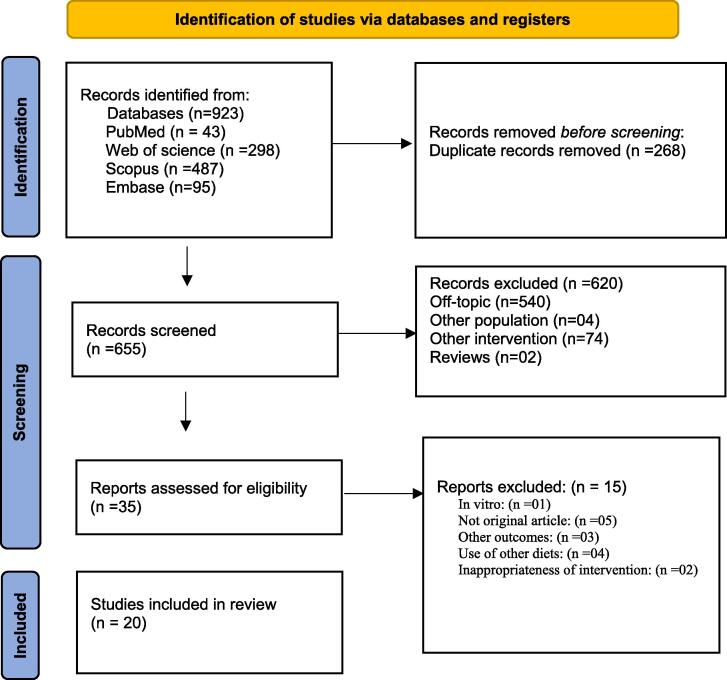
PRISMA flow diagram illustrating the results of database searches, the screening process, and the ultimate selection of studies for inclusion.

Initially, 923 references were retrieved from PubMed (Pánico et al., 2022), Scopus (487), Web of Science (298), and Embase (95). After deduplication, 35 articles were deemed eligible for inclusion. Ultimately, 20 studies meeting the inclusion criteria were included, while 15 were excluded (Fig. 1).

### Study characteristics

All included studies were original, experimental, and published in the English language. Most (85 %) were conducted between 2012 and 2024, with 15 % between 2000 and 2012. All utilized random sampling. The study population comprised primarily mice (90 %) and rats (10 %). Among mice, 80 % were male, 5 % were mixed-gender, and 15 % were unspecified; for rats, 5 % were male and 5 % were mixed-gender. Sample sizes were ≤50 in 40 % of the studies, >50 in 55 %, and unspecified in 5 %. Subject age averaged under 8 weeks in 35 % of studies, over 8 weeks in 55 %, and 15 % unspecified. Treatment duration was <20 weeks in 30 % of studies, ≥20 weeks in 55 %, and included both in 15 % (Table 1).

**Table 1:** Characteristics of article.

Characteristics of article			Number	Percent
Content Type of studies	Original article		20	100
Studies language	English		20	100
Type of Studies	Experimental study		20	100
Year of publication	2000–2012		3	15
	2012–2024		17	85
Type of sampling	Random sampling		20	100
Study population	Mouse	Male	16	80
		Male and Female	1	5
		Not mentioned	1	5
	Rat	Male	1	5
		Male and Female	1	5
		Not mentioned	0	0
Sample size	≤50		8	40
	>50		11	55
	Not mentioned		1	5
Age	< 8-week-old		7	35
	≥ 8-week-old		10	50
	Not mentioned		3	15
Treatment Duration	< 20 weeks		6	30
	≥ 20 weeks		11	55
	Both		3	15

### The results of risk of bias assessment

In this systematic review, 20 animal studies were included, examining the effects of a high-fat diet and arsenic on various organs. The risk of bias for each study was assessed using the SYRCLE's Risk of Bias Tool. This tool includes six main sections for identifying and evaluating the risk of bias in animal studies. In the Selection Bias section, all studies used valid randomization for allocating animals to treatment groups (low risk of bias). The Performance Bias assessment revealed that five studies employed group concealment in the histological sections (low risk of bias). Although this was not specified in other articles, standard conditions regarding animal housing, handling, and environmental factors such as temperature, light, and diet were standardized across all groups (moderate risk of bias). For Detection Bias, five studies fully complied with the required protocols (low risk of bias). Although other studies used standardized and objective methods for measuring and evaluating outcomes across all groups, there was no mention of blinded outcome assessment (moderate risk of bias). In terms of Attrition Bias, only one study fully adhered to the standards for handling bias from sample attrition (low risk of bias). In other studies, while all animals were included in the final analysis, no report was made regarding missing data (e.g., due to animal deaths) or the reasons for excluding any data or animals from the study (moderate risk of bias). The assessment of Reporting Bias showed that all studies reported all expected outcomes, including non-significant or negative findings, as well as predefined results and hypotheses (low risk of bias). Finally, the Other Bias assessment revealed that all studies adhered to the sample size standards for the number of animals used in laboratory studies and, in addition to using appropriate statistical analyses, disclosed any conflicts of interest or funding sources transparently (low risk of bias). Therefore, based on the evaluations, most of the studies included in this systematic review had appropriate design and execution, which helped reduce the risk of bias in the results. The most significant risk of bias was related to the lack of transparent reporting in processes such as group allocation concealment, blinded data analysis, and missing data in some studies. Overall, given the low impact of unreported issues in the articles, it can be concluded that the risk of bias in most studies was rated as “low.” However, there is still a need for improved reporting and procedural execution in future animal studies (Table 2 and Table 3.

**Table 2:** Risk of bias assessments.

**Table 3:** The characteristics of eligible and remained studies.

Number	Author	Publication year	Sample size	Study population	Aims	Experimental Methodology	Key finding	Treatment duration	Gender	Age	Type of sampling	Type of study
1	Zijun Y	2024	60	Mouse	To explore the fundamental mechanisms by addressing three critical areas: arsenic metabolism, liver pathology, and variations in signaling pathway expression.	In vivo	This study provides valuable insights into the mechanisms underlying liver damage resulting from sub chronic co-exposure to arsenic (As) and a high-fat diet (HFD), emphasizing the role of a healthy lifestyle in mitigating these effects.	12 weeks	Male	7-week-old	Random	Original article
2	Qiang S	2023	32–48	Rat	To analyze the combined impact of arsenic (As) and a high-fat diet (HFD) on vascular damage and uncover the underlying biological processes	In vivo	The combined exposure to As and HFD triggered pyroptosis in vascular endothelial cells through activation of the ASC/Caspase-1 signaling pathway.	13 weeks and 24 weeks	Male and Female	New born	Random	Original article
3	Calderón-DuPont D	2023	36	Mouse	To assess how concurrent exposure to HFD and arsenic affects fatty acid (FA) metabolism in white adipose tissue (WAT).	In vivo	1. Arsenic exposure exacerbated hyperinsulinemia induced by an HFD. 2. Arsenic worsened fatty acid metabolism dysfunction in white adipose tissue (WAT) in mice previously sensitized by an HFD.	16 weeks	Male	8-week-old	Random	Original article
4	Schiro G	2022	20	Mouse	To investigate how interactions between inorganic arsenic (iAs), normal diet (ND), HFD, and the NFE2L2/NRF2 gene influence gut microbiota composition and liver metabolism.	In vivo	The findings highlight how interactions between gut microbiota, diet, and arsenic intake contribute to the onset of metabolic disorders.	20 weeks	Male	8-week-old	Random	Original article
5	Carmean CM	2020	Not mentioned	Mouse	To examine the long-term consequences of arsenic exposure on fat accumulation and metabolic health in mice	In vivo	Arsenic exhibits anti-obesogenic properties, and its concentration at the source does not reliably predict its accumulation in tissues or associated phenotypic effects.	16 weeks	Male	7-week-old	Random	Original article
6	Zhang Y	2019	32	Mouse	To determine the effects of continuous arsenic exposure at environmentally relevant levels within an HFD framework on kidney disease progression.	In vivo	1. Arsenic exposure significantly amplified HFD-induced inflammatory responses and oxidative stress. 2. Male mice exhibited more severe physiological responses to HFD and arsenic exposure compared to female mice.	10 weeks and 24 weeks	Male and Female	New born	Random	Original article
7	Kalantari H	2019	40	Mouse	To assess whether arsenic contributes to HFD-induced liver damage using microarray-based analysis.	In vivo	This study demonstrated that arsenic in drinking water increased susceptibility to HFD-induced liver disease in mice by enhancing apoptosis-related pathways.	20 weeks	Male	Not mentioned	Random	Original article
8	Alboghobeish S	2019	48	Mouse	To investigate how prolonged exposure to arsenic and an HFD influences memory function and mitochondrial activity in the brain.	In vivo	1. Simultaneous exposure to an HFD and arsenic impaired memory function at least three times more severely than either exposure alone. 2. The neurotoxic effects may stem from mitochondrial oxidative stress and a reduction in the brain’s antioxidant capacity.	4, 8, 12, 16 or 20 weeks	Male	Not mentioned	Random	Original article
9	Ahangarpour A	2019	72	Mouse	To evaluate the toxic effects of long-term, simultaneous administration of arsenic and an HFD on the male reproductive system.	In vivo	Chronic exposure to HFD and arsenic led to hypogonadotropic hypogonadism, accompanied by reduced sperm count and testicular damage.	20 weeks	Male	Adult	Random	Original article
10	Zeinvand-Lorestani M	2018	76	Mouse	To study the relationship between chronic arsenic exposure and an HFD in driving liver toxicity, with a focus on the role of mitophagy.	In vivo	1. Alterations in the expression of Sqstm1, BNIP3, and Caspase-3 were associated with the extent of liver damage caused by combined exposure to arsenic and HFD. 2. The pro-apoptotic protein BNIP3 was linked to increased cell death induced by arsenic and HFD exposure.	20 weeks	Male	Adult	Random	Original article
11	Hemmati AA	2018	72	Mouse	To determine the extent to which an HFD alters arsenic-induced oxidative stress and lung tissue damage in mice.	In vivo	An HFD aggravated arsenic-induced lung damage through oxidative stress mechanisms.	20 weeks	Male	Adult	Random	Original article
12	Ahangarpour A	2018	72	Mouse	To examine the role of an HFD in exacerbating arsenic-induced cardiotoxicity in mice.	In vivo	1. Arsenic-induced cardiac injury was exacerbated by HFD consumption. 2. This effect may result from diminished antioxidant activity and/or increased oxidative stress and reactive oxygen species (ROS) production in cardiac tissues.	5 months	Male	Adult	Random	Original article
13	Ahangarpour A	2018	72	Mouse	To explore the effects of chronic HFD consumption on arsenic-induced oxidative damage in kidney tissues.	In vivo	HFD exposure intensified arsenic-induced oxidative stress and kidney damage.	20 weeks	Male	Adult	Random	Original article
14	Ahangarpour A	2018	72	Mouse	To investigate how arsenic exposure, combined with an HFD, contributes to diabetes development in male mice.	In vivo	Combined exposure to HFD and arsenic impaired oral glucose tolerance (OGTT) and pancreatic islet insulin secretion, likely due to mitochondrial oxidative stress.	20 weeks	Male	Adult	Random	Original article
15	Ahangarpour A	2018	72	Mouse	To assess the impact of long-term exposure to both an HFD and arsenic on thyroid function and lipid homeostasis.	In vivo	Co-exposure to HFD and arsenic induced hypothyroidism, characterized by reduced thyroid hormone levels, elevated plasma TSH, and enhanced T3 uptake, accompanied by hypolipidemia, hyperleptinemia, hyperadiponectinemia, oxidative stress, and reduced glutathione (GSH) levels.	20 weeks	Male	Adult	Random	Original article
16	Hou H	2017	40	Mouse	To examine how dietary fat influences arsenic-induced liver damage in C57BL/6 mice.	In vivo	This study underscores that an HFD may heighten the risk of arsenic-induced hepatotoxicity in regions with high arsenic contamination.	10 weeks	Male	5-week-old	Random	Original article
17	Dutta M	2014	24	Rat	To assess the combined effects of an HFD and arsenic exposure on cardiac and hepatic tissues in rats.	In vivo	Co-treatment with arsenic and an HFD aggravated arsenic-induced oxidative stress-mediated damage in cardiac and liver tissues.	8 days	Male	Not mentioned	Random	Original article
18	Tan M	2011	60	Mouse	To test the hypothesis that low-dose arsenic exposure increases liver sensitivity to damage in a mouse model of non-alcoholic fatty liver disease (NAFLD).	In vivo	These results suggest that even sub hepatotoxic levels of arsenic exposure can enhance HFD-induced liver toxicity.	10 weeks	Male	4-week-old	Random	Original article
19	Paul DS	2011	54–60	Mouse	To characterize the role of arsenic exposure and an HFD in promoting diabetes in weaned C57BL/6 mice.	In vivo	1. Arsenic exposure synergistically exacerbated HFD-induced obesity, contributing to glucose intolerance. 2. The diabetogenic effects of arsenic exposure appear to operate through mechanisms distinct from those driving obesity-induced type 2 diabetes.	20 weeks	Male	4-week-old	Random	Original article
20	Wu J	2008	60	Mouse	To evaluate how an HFD affects arsenic-induced liver fibrosis and the early stages of tumor development.	In vivo	Chronic exposure to inorganic arsenic led to liver injury, and HFD consumption significantly accelerated arsenic-induced hepatofibrosis.	10 months	Not mentioned	Adult	Random	Original article

## Discussion

### Diet and arsenic toxicity

**Fig. 2:**
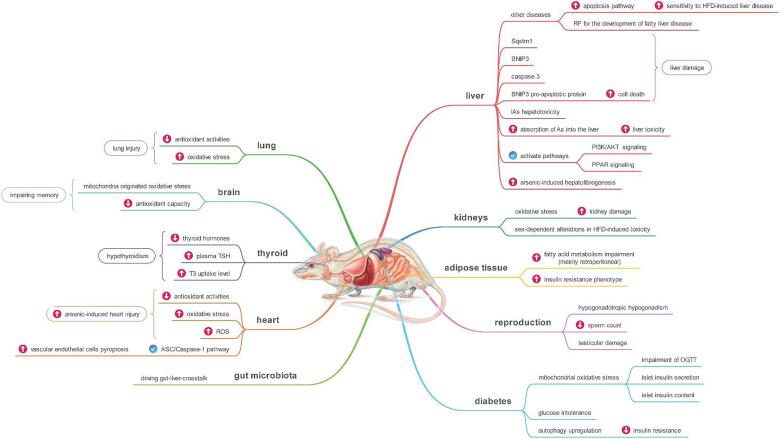
Mechanism of interactive effects of arsenic and high fat diet in various organs.

Diet significantly modulates arsenic bioavailability and toxicity (Yu et al., 2016). While some studies find no impact of dietary fiber on arsenic bioavailability or metabolism (Yager et al., 2015), others suggest dietary fat enhances the bioavailability of inorganic (iAsV) and organic (MMAs, DMAs) arsenic species, potentially via bile salt interactions (Pradeep Alava et al., 2013). One hypothesis posits that high-fat diets (HFDs), by supplying methyl groups, may accelerate the conversion of iAs to MMAs and DMAs, thereby promoting arsenic elimination (Silva, 2016). However, this contradicts numerous studies showing that HFDs can also increase arsenic bioavailability (Dey et al., 2020). Furthermore, HFDs induce oxidative stress in various organs, including the brain, heart, testes, and kidneys, potentially leading to additive or synergistic toxic effects when combined with arsenic exposure (Abha Sharma and Flora, 2018, Kesh et al., 2016). Ongoing research aims to clarify these organ-specific mechanisms (Table 4;Fig. 2 ).

**Table 4:** The effect of high-fat diet on arsenic toxicity in various organs.

Dysfunction of various organs	Animals	Concentration of As-HFD	Time	Remarks and comment	Reference
Adipose tissue	C57BL/ 6male mice	HFD (40 % kcal fat) with arsenic exposure through drinking water (100 μg/L)	16 weeks	Arsenic in sensitized mice by HFD worsens fatty acid metabolism impairment in WAT, mainly retroperitoneal, along with an exacerbated insulin resistance phenotype.	(Calderón-DuPont et al., 2023) https://doi.org/10.1016/j.taap.2023.116428
Brain	NMRI/ male mice	HFD (58 % kcal fat) with arsenic exposure through drinking water (25 or 50 ppm)	20 weeks	Simultaneous exposure to HFD and As impaired memory at least three times more than exposure to each alone. These toxic effects may be due to mitochondrial-originated oxidative stress, accompanied by depleted antioxidant capacity.	(Alboghobeish et al., 2019) https://doi.org/10.1007/s11011–019–00467–4
Diabetes	NMRI/ male mice	HFD (58 % kcal fat) with arsenic exposure through drinking water (25 or 50 ppm)	20 weeks	HFD and arsenic concomitant administration induced impairment of OGTT and islet insulin secretion or content through mitochondrial oxidative stress.	(Ahangarpour et al., 2018) https://doi.org/10.22037/ijpr.2018.2174
	C57BL/ 6male mice	HFD (58 % kcal fat) with arsenic exposure through drinking water (25 or 50 ppm)	20 weeks	As exposure acts synergistically with HFD in producing glucose intolerance. However, the mechanisms of the diabetogenic effects of As exposure differs from the mechanisms associated with type 2 diabetes.	(Paul et al., 2011) https://doi.org/10.1289/ehp.1002877
	NMRI/ male mice	HFD (58 % kcal fat) with arsenic exposure through drinking water (25 or 50 ppm)	20 weeks	Reduced insulin resistance in HFD-As-induced diabetes may be mediated by autophagy upregulation.	(Zeinvand-Lorestani et al., 2018) https://doi.org/10.1038/s41598–018–30323–5
Gut microbiota	C57BL/ 6male mice Nrf2+/+ and Nrf2-/- mice	HFD (60 % kcal fat) with arsenic exposure through drinking water (25 ppm).	20 weeks	The importance of the microbial community in driving gut-liver-cross talk during iAs and HFD exposure.	(Schiro et al., 2022) https://doi.org/10.3389/fmicb.2022.1041188
Heart	NMRI/ male mice	HFD (58 % kcal fat) with arsenic exposure through drinking water (25 or 50 ppm)	20 weeks	HFD increased arsenic-induced heart injury. This effect may be due to a reduction in antioxidant activity and/or an increase in oxidative stress and ROS in mouse heart tissues.	(Ahangarpour et al., 2018) https://doi.org/10.4103/1735–5362.226242
	Male Wistar rat	HFD (50 % kcal fat) with arsenic 0.325, 0.75, 1.5 mg/kg body weight (i.p)	8 days	A high-fat diet increased arsenic-induced oxidative stress.	(Dutta et al., 2014) https://doi.org/10.1016/j.fct.2013.12.019
	Male Wistar rat			The combination of As and HFD induced Vascular endothelial cells pyroptosis through activation of the ASC/Caspase-1 pathway.	(Su et al., 2023) https://doi.org/10.1016/j.tox.2023.153691
Kidneys	C57BL/ 6male mice	HFD (58 % kcal fat) with arsenic exposure through drinking water (100 ppm)	13 and 24 weeks	Arsenic exposure causes sex-dependent alterations in HFD-induced toxicity.	(Zhang et al., 2019) https://doi.org/10.1016/j.cbi.2019.06.032
	NMRI/ male mice	HFD (58 % kcal fat) with arsenic exposure through drinking water (25 or 50 ppm)	20 weeks	HFD increased arsenic-induced kidney damage through oxidative stress in mice.	(Ahangarpour et al., 2018)
Liver	NMRI/ male mice	HFD (58 % kcal fat) with arsenic exposure through drinking water (25 or 50 ppm)	20 weeks	Drinking water could increase sensitivity in mice to HFD-induced liver disease by strengthening the apoptosis pathway.	(Kalantari et al., 2019) DOI: 0.1007/s11356–019–05995–2
	C57BL/ 6male mice	HFD (42 % kcal fat) with arsenic exposure through drinking water (4.9 ppm)	10 weeks	Sub hepatotoxic arsenic exposure enhances the toxicity of HFD. Arsenic exposure might be a risk factor for the development of fatty liver disease in human populations.	(Tan et al., 2011) https://doi.org/10.1016/j.taap.2011.06.013
	NMRI/ male mice	HFD (58 % kcal fat) with arsenic exposure through drinking water (25 or 50 ppm)	20 weeks	The changes observed in the expression of Sqstm1, BNIP3, and caspase three can be related to the level of liver damage caused by exposure to arsenic and HFD. It is likely that BNIP3, a pro-apoptotic protein, is associated with increased cell death.	(Zeinvand-Lorestani et al., 2018) https://doi.org/10.1007/s11356–018–3484–3
	C57BL/ 6male mice	HFD (54 % kcal fat) with arsenic exposure through drinking water (3 mg/L)	10 weeks	HFD increases the sensitivity of mice to iAs in drinking water, leading to enhanced hepatotoxicity. HFD might enhance the risk of iAs hepatotoxicity in iAs-polluted regions.	(Tan et al., 2011) https://doi.org/10.1016/j.taap.2011.09.019
	C57BL/ 6male mice	HFD (54 % kcal fat) with arsenic exposure through drinking water (0.05–50 mg/L)	12 weeks	A high-fat diet increased the absorption of As into the liver and enhanced liver toxicity, which became progressively more severe as the As concentration increased. Co-exposure to a high-fat diet and As (III) activated PI3K/AKT and PPAR signaling as well as fatty acid metabolism pathways. Additionally, the expression of proteins related to lipid cell function, lipid metabolism, and body weight regulation was also affected.	(Ye et al., 2024) https://doi.org/10.1002/tox.24037
Liver fibrosis	Kunming white mice	sodium arsenide (200 ppm) or sodium arsenate (200 ppm) in the drinking water, and a diet containing 20 % added fat.	10 months	Chronic inorganic arsenic exposure in mice produces liver injury, and a high-fat diet markedly increases arsenic-induced hepatofibrogenesis.	(Wu et al., 2008) https://doi.org/10.3181/0707–MR–203
Lung	NMRI/ male mice	HFD (58 % kcal fat) with arsenic exposure through drinking water (25 or 50 ppm)	20 weeks	Chronic arsenic exposure produced lung injury, and HFD enhanced it in mice. This effect is explained by the reduced antioxidant activities and/or increased oxidative stress in the mice's lung tissue.	(Hemmati et al., 2018) https://doi.org/10.4081/monaldi.2018.903
Obesity	C57BL/ 6male mice	HFD (60 % kcal fat) with arsenic exposure through drinking water (50 ppm)	8 weeks	Arsenic is obesogenic, and concentration at the source poorly predicts arsenic accumulation and phenotypic outcomes. In future studies, investigators should consider the internal accumulation of arsenic rather than source concentration when assessing the outcomes of arsenic exposure.	(Carmean et al., 2020) https://doi.org/10.1002/oby.22770
Reproduction	NMRI/ male mice	HFD (58 % kcal fat) with arsenic exposure through drinking water (25 or 50 ppm)	20 weeks	Chronic exposure to HF and arsenic-induced hypogonadotropic hypogonadism concomitant with sperm count reduction and testicular damage.	(Ahangarpour et al., 2019) https://doi.org/10.18502/jfrh.v13i4.2645
Thyroid	NMRI/ male mice	HFD (58 % kcal fat) with arsenic exposure through drinking water (25 or 50 ppm)	20 weeks	Combined exposure to HFD and arsenic-induced hypothyroidism via reduction of thyroid hormones and enhancement of plasma TSH and T3 uptake levels concomitant with hypolipidemia, hyperleptinemia, hyperadiponectinemia, induction of oxidative stress, and reduction of GSH level.	(Ahangarpour et al., 2018) DOI https://doi.org/10.1007/s12011-017–1068-1

### Role of fat type and nutrients in modulating arsenic toxicity

Emerging evidence suggests that the type of dietary fat plays a crucial role in mitigating arsenic toxicity (Bibha et al., 2024). Saturated fatty acids (SFAs) have been linked to increased oxidative stress, inflammation, and gut permeability, which can exacerbate arsenic’s toxic effects (Bibha et al., 2024, Yu et al., 2016). In contrast, specific polyunsaturated fatty acids (PUFAs), particularly omega-3 fatty acids, exert anti-inflammatory effects and help maintain membrane integrity and mitochondrial function, potentially offering protective effects against arsenic toxicity (Xu et al., 2025, Pace et al., 2017). Additionally, dietary components such as antioxidants (e.g., vitamin C, vitamin E, and polyphenols) and fiber may further mitigate arsenic-induced damage by reducing oxidative stress and modulating the gut microbiota (Renu et al., 2023, Bjørklund et al., 2022). These findings suggest that the quality of dietary fat and overall nutrient composition are important considerations in understanding the dietary modulation of arsenic toxicity.

#### Arsenic, High-Fat Diet, and obesity Incidence

The relationship between arsenic exposure and BMI or obesity is complex and conflicting (Farkhondeh et al., 2019). While some human and animal studies suggest obesity or elevated BMI may increase susceptibility to arsenic-related diseases, arsenic's direct impact on these parameters remains unclear. One mouse study found increased body weight in female mice exposed to arsenic on a low-fat diet but not in males; this effect was absent in HFD-fed females, while HFD-fed males gained weight irrespective of arsenic exposure (Zhang et al., 2019). Conversely, another study noted that male mice exposed to both arsenic and HFD gained less weight than those on HFD alone (Ahangarpour et al., 2018).

Furthermore, rat research has shown a negative correlation between hepatic arsenic levels and weight gain, fat mass, and hepatic triglyceride accumulation. In HFD-fed mice, arsenic exposure reduced weight gain, fat mass, and hepatic triglycerides compared to HFD alone, with no such effect observed in standard-diet mice. Arsenic exposure did not alter activity levels in either group. These findings emphasize considering tissue-level arsenic accumulation, rather than ingested arsenic, as a more accurate metric for assessing arsenic's metabolic effects and improving the predictability of future research (Carmean et al., 2020).

#### Arsenic, high-fat diet, and fatty acid metabolism in adipose tissue

Dysregulated fatty acid metabolism in white adipose tissue, often triggered by excessive calorie intake or exposure to endocrine-disrupting chemicals, is crucial in the development of obesity and insulin resistance (Pánico et al., 2022). Research on combined arsenic and HFD effects in mice revealed that arsenic exposure with an HFD increases serum markers of selective insulin resistance, promotes fatty acid re-esterification in adipose tissue, and diminishes lipolysis (Paul et al., 2011). These effects are most pronounced in retroperitoneal adipose tissue. Specifically, the combination of arsenic and HFD, versus HFD alone, leads to increased fat mass, larger adipocytes, elevated triglyceride content, and reduced fasting-stimulated lipolysis in retroperitoneal adipose tissue. This reduced lipolysis correlates with decreased phosphorylation of HSL and perilipin. At the gene expression level, arsenic exposure in HFD-fed mice downregulates genes for fatty acid uptake (LPL, CD36), oxidation (PPARα, CPT1), lipolysis (ADRβ3), and glycerol transport (AQP7, AQP9) (Calderón-DuPont et al., 2023). While combined arsenic and HFD resulted in only modest increases in weight gain and feed efficiency, it exacerbated HFD-induced hyperinsulinemia (Ahangarpour et al., 2018). These findings suggest arsenic exposure in mice predisposed to metabolic dysfunction by an HFD worsens fatty acid metabolism disorders in white adipose tissue, particularly retroperitoneal, contributing to a more severe insulin-resistant state.

#### Arsenic, High-Fat Diet, and the Incidence of diabetes

Mice on a high-fat diet (HFD) typically exhibit characteristics of type 2 diabetes, including increased weight and fat, elevated fasting glucose and insulin levels, impaired glucose tolerance, and pronounced insulin resistance compared to mice on a low-fat diet. Interestingly, mice exposed to both arsenic and an HFD accumulated less fat and had lower serum triglycerides, fasting glucose, and insulin than those on HFD alone. However, these mice displayed more significant glucose intolerance than the HFD-only group. These findings suggest that while inorganic arsenic can mitigate diet-induced obesity in mice, it synergistically interacts with an HFD to induce glucose intolerance, a prediabetes hallmark. This combination of glucose intolerance, elevated blood glucose, and near-normal insulin levels differs from the typical type 2 diabetes phenotype (Paul et al., 2011).

Similar results have been reported. One study observed that HFD-fed mice showed increased fat accumulation, elevated fasting glucose, and greater insulin resistance compared to low-fat diet controls. Arsenic administration dose-dependently mitigated these diabetic indices. Despite these improvements, glucose intolerance remained elevated in arsenic-treated HFD-fed rats. This suggests that arsenic exposure interacts with HFD-induced obesity to increase glucose intolerance, potentially through mechanisms distinct from those typically associated with obesity-related type 2 diabetes (Barrett, 2011). Another study confirmed these findings, demonstrating that arsenic exposure in HFD-fed mice reduced fasting insulin, insulin resistance, β-cell dysfunction, and systemic insulin resistance (Carmean et al., 2020). Although weight gain was less pronounced in mice exposed to both HFD and arsenic, arsenic enhanced the hyperinsulinemia and subsequent insulin resistance induced by the HFD (Calderón-DuPont et al., 2023).

A 2018 study further explored underlying mechanisms. HFD-fed mice exhibited increased fasting glucose, impaired glucose and insulin tolerance, dyslipidemia, elevated leptin, β-amylase, hepatic mitochondrial oxidative stress, and liver enzymes, alongside decreased fasting insulin, β-cell activity, adiponectin, and islet insulin secretion. However, adding arsenic to the HFD regimen reduced fasting glucose, fasting insulin, insulin resistance and tolerance, β-cell function, dyslipidemia, and islet insulin secretion. This combined exposure also increased glucose tolerance, leptin, adiponectin, hepatic mitochondrial oxidative stress, and liver enzymes. This distinct form of diabetes, characterized by impaired glucose tolerance and islet insulin secretion without typical hyperglycemia, hyperinsulinemia, and insulin resistance of type 2 diabetes, is accompanied by pancreatic β-cell failure, hepatotoxicity, hyperleptinemia, hyperlipidemia, hypoadiponectinemia, and hepatic oxidative stress and damage (Ahangarpour et al., 2018). A recent review highlighted that arsenic-induced diabetes in mice primarily manifests as glucose intolerance, with arsenic exacerbating HFD-induced glucose intolerance. However, the mechanisms of arsenic-induced diabetes differ from those of HFD-induced type 2 diabetes (Wang and Zhao, 2022). Finally, one study investigated the potential connection between insulin resistance and obesity through autophagy. This study showed that chronic HFD feeding, arsenic exposure in mouse livers, and increased oxidative stress led to autophagy restoration and modulation of genes involved in autophagosome formation. The authors suggest that arsenic's role in inducing autophagy may explain the reduced insulin resistance in HFD-induced diabetes with arsenic (Zeinvand-Lorestani et al., 2018).

#### Arsenic, High-Fat Diet, and liver disorders

Several studies have investigated the combined impact of arsenic and HFD on liver damage. One study confirmed HFD-induced steatohepatitis via histological evaluation and elevated plasma ALT and AST levels. While arsenic alone did not affect liver damage markers in low-fat diet animals, it significantly exacerbated liver damage in those consuming an HFD. This effect was associated with increased inflammation and fibrin extracellular matrix (ECM) deposition, suggesting that the combination of arsenic exposure and HFD may be a risk factor for fatty liver disease development in humans (Tan et al., 2011). HFDs can also alter arsenic uptake and excretion, increasing arsenic-induced hepatic DNA damage and affecting cell death, growth, signal transduction, lipid metabolism, and insulin signaling pathways. Compared to gene expression profiles with arsenic or HFD alone, the insulin signaling pathway significantly mediates the interactive effects of these two factors. HFD consumption can increase rats' sensitivity to arsenic in drinking water, thereby increasing hepatotoxicity. Consequently, HFDs may increase the risk of arsenic-induced liver damage in arsenic-contaminated areas (Hou et al., 2017).

Another study found that caspase-8, a key mediator in apoptosis, is crucial in apoptosis induced by combined arsenic and HFD. This research indicated that arsenic increases susceptibility to HFD-induced liver disease by enhancing apoptosis (Kalantari et al., 2019). A more recent study examining liver damage mechanisms from combined arsenic exposure and HFD observed that HFD increased arsenic uptake in the liver, leading to increased hepatotoxicity that worsened with rising arsenic concentration. Co-exposure to arsenic and HFD induces PI3K/AKT and PPAR activation and signaling, along with fatty acid metabolism pathway activation. Furthermore, the expression of proteins involved in lipid cell function, lipid metabolism, and body weight regulation is also affected (Ye et al., 2024).

Another study investigated changes in Sqstm1, mitophagy (BNIP3), and apoptosis (caspase 3) gene expression in the livers of mice exposed to cytotoxic arsenide concentrations in drinking water while consuming an HFD. Mitochondrial destruction via mitophagy can lead to apoptosis during lipotoxicity. Both lipotoxicity and arsenic-induced cytotoxicity can alter mitophagy and apoptosis. The Sqstm1-derived protein, p62, is vital for hepatic energy homeostasis and, through its influence on mTOR, MAPK, and NF-κB signaling, may help regulate autophagic responses. This study showed decreased BNIP3 expression in groups exposed to either arsenic or HFD alone but a significant increase in the combined exposure group. P62 gene expression also decreased in single-exposure groups but increased in the combined-exposure group. Caspase 3 expression, a key factor in apoptotic liver cell death, was elevated in all groups, with the highest levels in the combined-exposure group. These results suggest that changes in Sqstm1, BNIP3, and caspase-3 expression may relate to the extent of liver damage caused by combined exposure to arsenic and an HFD (Zeinvand-Lorestani et al., 2018).

#### Arsenic, high-fat diet, and incidence of liver fibrosis

A study investigated the impact of a high-fat diet (HFD) on arsenic-induced liver fibrosis. Serum AST levels, a liver damage marker, were elevated in arsenic-exposed mice, with HFD further increasing these levels. Histopathological analysis revealed liver inflammation, steatosis, hepatocyte degeneration, and fibrosis from arsenic alone, but HFD significantly exacerbated these effects. Combined exposure synergistically increased pro-inflammatory genes (TNF-α, IL-6, iNOS, chemokines, and macrophage inflammatory protein-2), the stress-related gene oxygenase-1, and fibrosis-associated genes (procollagen-1 and −3, SM-actin, and TGF-β). Conversely, metallothionein-1 and GSH S-transferase-pi expression decreased. Genes encoding matrix metalloproteinases (MMP2, MMP9) and tissue inhibitors of metalloproteinases (TIMP1, TIMP2), indicating early carcinogenic events, also increased. In summary, chronic arsenic exposure induces liver injury, and an HFD significantly potentiates arsenic-induced hepatofibrogenesis (Wu et al., 2008).

#### Arsenic, high-fat diet, and the gut microbiota-liver metabolism relationship

A study explored the interplay between diet, NFE2L2/NRF2 gene expression, and gut microbiota in chronic inorganic arsenic (iAs) exposure and its liver effects in mice fed normal or HFD. Chronic arsenic and HFD exposure disrupted gut microbial activity and altered the host gut microbial community, differentially affecting bacteria and influencing host health. These changes in gut microbial activity may alter liver metabolic activity. The study suggests that interactions among gut microbes, diet, and arsenic intake contribute to the development of metabolic liver disease (Schiro et al., 2022).

#### The effect of arsenic and high-fat diet on the cardiovascular system

One study investigated arsenic exposure (25 or 50 ppm) combined with an HFD or low-fat diet for five months in rats, assessing body weight, heart weight to body weight ratio, cardiac biochemical markers (CPK, LDH, AST, ALT), lipid profile (triglycerides, cholesterol, LDL, VLDL, HDL), and cardiac histology. Arsenic alone significantly decreased cardiac glutathione levels and catalase activity, coupled with increased reactive oxygen species (ROS), malondialdehyde, and biochemical enzyme levels (Ahangarpour et al., 2018).

HFD alone induced similar changes, including an increase in lipid profile. However, both alone and in combination with HFD, arsenic exposure decreased most lipid profile factors, except for HDL. The study found that HFD exacerbated arsenic-induced cardiac injury, possibly due to reduced antioxidant activity and/or increased oxidative stress and ROS in cardiac tissue (Ahangarpour et al., 2018). Other research similarly shows that HFDs can increase arsenic-induced oxidative stress (Dutta et al., 2014).

Another study examined combined arsenic and HFD effects on vascular health in mice. Simultaneous exposure significantly increased serum lipid levels and aortic fat accumulation compared to either agent alone. This combined exposure also altered blood pressure and disrupted the morphology of the abdominal aorta. Furthermore, it increased the expression of vascular endothelial cell pyroptosis proteins (ASC, Pro-caspase-1, Caspase-1, IL-8, and IL-1β) and vascular endothelial adhesion factors (VCAM-1 and ICAM-1). Vascular damage increased with more prolonged exposure to both arsenic and HFD, with more severe damage in female rats. This study suggested that arsenic- and HFD-induced vascular endothelial cell pyroptosis via ASC/Caspase-1 pathway activation may be a molecular mechanism for arsenic-induced vascular injury associated with HFD exposure (Su et al., 2023).

#### The effect of arsenic and high-fat diet on kidney function

High-fat diets (HFDs) exacerbate arsenic-induced kidney damage. Chronic exposure to arsenic (25 or 50 ppm) or HFD alone decreased the kidney weight to body weight ratio, with this decrease being more pronounced when both were consumed concurrently. Similarly, chronic exposure to 50 ppm arsenic decreased total protein and albumin and increased plasma urea and creatinine; these effects were amplified when combined with HFD. Histopathological analysis revealed that combined exposure increased inflammatory cell infiltration, cellular swelling, and loss of the brush border while decreasing the number of tubular cells and the glomerular diameter. While HFD alone had minimal effects on oxidant/antioxidant markers (GSH, ROS, MDA), its concurrent consumption with arsenic increased ROS and MDA levels and significantly decreased GSH levels. These findings suggest that HFD potentiates arsenic-induced kidney damage in mice by increasing oxidative stress (Ahangarpour et al., 2018).

Another study investigated lifetime arsenic exposure and HFD on renal pathogenesis. Mice exposed to 100 ppb arsenic from before conception, with offspring fed either a regular or HFD, showed that arsenic significantly increased HFD-induced glomerular area expansion, mesangial matrix accumulation, and fibrosis compared to controls. HFD alone increased renal inflammation and fibrosis (elevated IL-1β, ICAM-1, fibronectin), but arsenic significantly enhanced these HFD-induced oxidative stress and inflammatory responses. Male mice generally exhibited more severe responses to HFD and arsenic than females, indicating arsenic induces sex-dependent changes in HFD-induced kidney injury (Zhang et al., 2019).

#### Arsenic, high-fat diet, and thyroid function

The thyroid gland plays a crucial role in metabolism and adipose tissue function. Chronic HFD consumption is associated with increased T3, TSH, leptin, lipids, ROS, and MDA while decreasing lipoprotein, albumin, adiponectin, and GSH. Chronic arsenic exposure reduces T3 and GSH while increasing TSH, leptin, ROS, MDA, and the T4/T3 ratio. Combined exposure to both HFD and arsenic further altered the lipid profile, T4, albumin, and total protein levels while increasing TSH, adiponectin, and leptin and decreasing the T4/T3 ratio. Additionally, it increased ROS and MDA. These findings suggest that simultaneous HFD and arsenic exposure can induce hypothyroidism, characterized by decreased thyroid hormones, increased plasma TSH and T3 uptake, hypolipidemia, hyperleptinemia, hyperadiponectinemia, oxidative stress, and decreased GSH (Ahangarpour et al., 2018).

#### Arsenic, high-fat diet, and lung function

The combined effects of HFDs and arsenic on lung function are sparsely studied. Chronic arsenic exposure (25 or 50 ppm) significantly decreased glutathione and catalase activity in lung tissue while increasing ROS, malondialdehyde, and nitrite levels; however, it did not affect superoxide dismutase activity or hydroxyproline content. HFD consumption increased arsenic accumulation in lung tissue. Furthermore, chronic HFD consumption exacerbates all markers of arsenic-induced lung damage, likely by increasing oxidative stress through decreased antioxidant activity and/or increased ROS in the lungs (Shu et al., 2025). Collagen deposition, indicative of pulmonary inflammation and potential fibrosis, assessed by hydroxyproline levels, increased in lung tissue with simultaneous chronic HFD and arsenic consumption, suggesting pulmonary inflammation and possible fibrosis. This combined exposure also causes lung tissue congestion, inflammation, and alveolar wall degeneration (Hemmati et al., 2018).

#### Arsenic, high-fat diet, and brain function

One study evaluated the long-term combined effects of arsenic and a high-fat diet (HFD) on brain function and mitochondria in male mice. Mice exposed to 25 or 50 ppm arsenic in drinking water and/or HFD for up to 20 weeks showed memory impairment (reduced retention latency) with arsenic or HFD alone after 12 and 16 weeks, respectively. Combined exposure accelerated this impairment to 8 weeks. HFD alone increased mitochondrial membrane damage, ROS, and MDA while reducing GSH. Co-administration of HFD and arsenic amplified these toxic effects. In conclusion, the combination of HFD and arsenic impaired memory at least three times more severely than either factor alone, likely through mitochondrial-induced oxidative stress or reduced brain antioxidant capacity (Alboghobeish et al., 2019).

#### Arsenic, high-fat food, and reproduction

Research indicates that HFDs can increase testicular weight and volume while reducing the number of interstitial cells and vacuoles in the germinal epithelium. Chronic arsenic exposure is associated with elevated plasma estradiol levels, a decreased testosterone-to-estradiol ratio, and a reduced sperm count. The combination of arsenic and HFD further reduces plasma LH, FSH, and testosterone levels and exacerbates tissue damage. This combined exposure exerts reproductive toxic effects, potentially through functional hypogonadism and hypogonadotropic hypogonadism, leading to decreased gonadotropin and testosterone and, consequently, reduced sperm count. Furthermore, combined exposure can induce testicular tissue changes, including germ cell degeneration vacuolation and separation of the seminiferous tubules (Ahangarpour et al., 2019).

Although numerous studies have demonstrated that a high-fat diet (HFD) potentiates arsenic-induced toxicity via mechanisms such as increased oxidative stress, inflammation, and metabolic dysregulation, some reports suggest that this interaction may be more nuanced and potentially dose-dependent. For instance, Carmean et al. (Carmean et al., 2020) observed that low-dose arsenic exposure in combination with HFD reduced adiposity and improved metabolic parameters, which may reflect the activation of adaptive cellular pathways, such as enhanced AMPK signaling or improved insulin sensitivity. Similarly, Ahangarpour et al. (Ahangarpour et al., 2018) reported improvements in lipid profiles in arsenic-exposed animals under HFD conditions, suggesting potential compensatory mechanisms. These findings highlight the possibility of biphasic or hormetic responses, where low doses of arsenic, particularly in metabolically active tissues such as adipose tissue, may transiently activate protective pathways (Ro et al., 2022). Several factors, including the dose and duration of arsenic exposure, the type of fat in the diet, and organ-specific susceptibilities, likely contribute to these dual effects (Martínez-Castillo et al., 2021). These considerations underscore the importance of carefully interpreting the arsenic-HFD interaction, particularly in studies employing varying exposure protocols.

## Conclusion

This systematic review provides compelling evidence that high-fat diets significantly exacerbate the toxicological effects of arsenic exposure. Our findings suggest that the co-occurrence of a high-fat diet and arsenic exposure increases arsenic bioavailability, resulting in elevated oxidative stress and widespread damage across multiple organ systems, including the brain, cardiovascular system, kidneys, and reproductive organs. Several mechanistic pathways likely underlie this interaction. High-fat diets may alter bile acid metabolism, leading to increased arsenic absorption and systemic circulation. Additionally, the combined impact of dietary fat and arsenic disrupts key metabolic pathways—particularly lipid metabolism in adipose tissue—thereby contributing to insulin resistance and glucose dysregulation, even in the absence of classical type 2 diabetes markers. Emerging evidence also suggests that dietary fat modulates hepatic autophagy, linking arsenic toxicity to a broader spectrum of metabolic dysfunction. Given arsenic’s unique diabetogenic potential, these findings have significant public health implications, particularly for individuals consuming high-fat diets in areas contaminated with arsenic. Adopting diets low in saturated fats and rich in polyunsaturated fats, fiber, and antioxidants may help mitigate these risks. This review emphasizes the importance of considering dietary patterns in risk assessments of arsenic exposure. Future research should investigate molecular mechanisms, dose–response relationships, and tissue-specific effects to inform evidence-based dietary recommendations and targeted public health interventions.

## CRediT authorship contribution statement

**Hoda Vahedi:** Conceptualization, Methodology, Investigation, Data curation, Writing – original draft. **Fateme Pourmotahari:** Formal analysis, Data curation, Writing – review & editing. **Ali Akbar Oroojan:** Formal analysis, Data curation, Writing – review & editing. **Amin Rasekhian:** Investigation, Visualization, Resources. **Soheila Alboghobeish:** Conceptualization, Supervision, Project administration, Funding acquisition, Writing – review & editing.

## Declaration of competing interest

The authors declare that they have no known competing financial interests or personal relationships that could have appeared to influence the work reported in this paper.

## Data Availability

Data will be made available on request.
